# Bioinspired Design of 3D-Printed Cellular Metamaterial Prosthetic Liners for Enhanced Comfort and Stability

**DOI:** 10.3390/biomimetics9090540

**Published:** 2024-09-06

**Authors:** Vasja Plesec, Gregor Harih

**Affiliations:** Laboratory for Integrated Product Development and CAD, Faculty of Mechanical Engineering, University of Maribor, 2000 Maribor, Slovenia; gregor.harih@um.si

**Keywords:** bioinspired design, metamaterial model, cellular structure, additive manufacturing, lower-limb prosthetic, 3D printing, finite element method

## Abstract

Traditional prosthetic liners are often limited in customization due to constraints in manufacturing processes and materials. Typically made from non-compressible elastomers, these liners can cause discomfort through uneven contact pressures and inadequate adaptation to the complex shape of the residual limb. This study explores the development of bioinspired cellular metamaterial prosthetic liners, designed using additive manufacturing techniques to improve comfort by reducing contact pressure and redistributing deformation at the limb–prosthesis interface. The gyroid unit cell was selected due to its favorable isotropic properties, ease of manufacturing, and ability to distribute loads efficiently. Following the initial unit cell identification analysis, the results from the uniaxial compression test on the metamaterial cellular samples were used to develop a multilinear material model, approximating the response of the metamaterial structure. Finite Element Analysis (FEA) using a previously developed generic limb–liner–socket model was employed to simulate and compare the biomechanical behavior of these novel liners against conventional silicone liners, focusing on key parameters such as peak contact pressure and liner deformation during donning, heel strike, and the push-off phase of the gait cycle. The results showed that while silicone liners provide good overall contact pressure reduction, cellular liners offer superior customization and performance optimization. The soft cellular liner significantly reduced peak contact pressure during donning compared to silicone liners but exhibited higher deformation, making it more suitable for sedentary individuals. In contrast, medium and hard cellular liners outperformed silicone liners for active individuals by reducing both contact pressure and deformation during dynamic gait phases, thereby enhancing stability. Specifically, a medium-density liner (10% infill) balanced contact pressure reduction with low deformation, offering a balance of comfort and stability. The hard cellular liner, ideal for high-impact activities, provided superior shape retention and support with lower liner deformation and comparable contact pressures to silicone liners. The results show that customizable stiffness in cellular metamaterial liners enables personalized design to address individual needs, whether focusing on comfort, stability, or both. These findings suggest that 3D-printed metamaterial liners could be a promising alternative to traditional prosthetic materials, warranting further research and clinical validation.

## 1. Introduction

Each year, over 200,000 lower-limb amputations occur in Europe, a number rising due to age-related conditions such as vascular disease, cancer, infections, and tissue damage [1]. These amputations significantly impact patients’ mobility, workability, social engagement, and independence [2]. Health professionals provide custom-made prostheses to restore mobility and reintegrate patients into society [3,4]. Advances in biomechanics, prosthesis design, and manufacturing have improved prosthesis fit, reduced pain, decreased tissue and joint stress, enhanced gait, lowered metabolic costs, and improved esthetics [5,6]. However, the manufacturing of prosthetic sockets still relies heavily on the prosthetist’s expertise [4]. Assessing the fit, comfort, and stability of a prosthesis is difficult to quantify, leading to a trial-and-error process that is time-consuming, wasteful, and expensive [7]. Consequently, creating a prosthesis that effectively distributes load, provides stability, and ensures safety remains challenging. In the first year after amputation, patients need about nine visits for adjustments, and nearly 20% of amputees receive a new prosthesis annually due to fit issues [8].

Research has shown that soft tissue exhibits rapid deformation under small loads, while its stiffness experiences an exponential increase as the load magnitude increases [9]. This behavior proves advantageous, as it facilitates the redistribution of contact pressure across wider areas, diminishing stress concentration and averting tissue damage. However, complications arise when the load surpasses the tissue’s deformation capacity, particularly in regions featuring a thin layer of soft tissue, such as the tibial crest or fibular head. In such cases, discomfort and pain arise, potentially resulting in tissue and bone impairment. The pain threshold and pain tolerance are influenced by numerous factors, including the limb’s condition, topological and morphological attributes, the psychological state of the patient, and pain sensitivity, among others [10]. Consequently, establishing a universal pain threshold becomes impracticable.

Limited research has been conducted on the assessment of pain thresholds and pain tolerance specifically in the context of transtibial residual limbs, utilizing an indentation test [10,11,12,13,14]. Pain tolerance, as defined in the existing literature, refers to the minimum average pressure required to trigger pain, whereas pain tolerance denotes the maximum average pressure that an individual can endure without excessive effort. Lee et al. reported a range of pain thresholds spanning from 350 kPa to 710 kPa, depending upon the site on the residual limb and the material constituting the indenter interface [10]. In contrast, pain tolerances yielded considerably higher values, ranging from 600 kPa to 1140 kPa. In another study conducted by Wu et al., employing a similar methodology for pain threshold and pain tolerance, significantly lower values were also found for pain threshold (ranging from 369.3 ± 154.5 kPa to 919.6 ± 161.7 kPa) compared to pain tolerance (ranging from 547.6 ± 109.1 kPa to 1158.3 ± 203.2 kPa), with variations arising depending on the measurement site [12]. 

**Figure 1:**
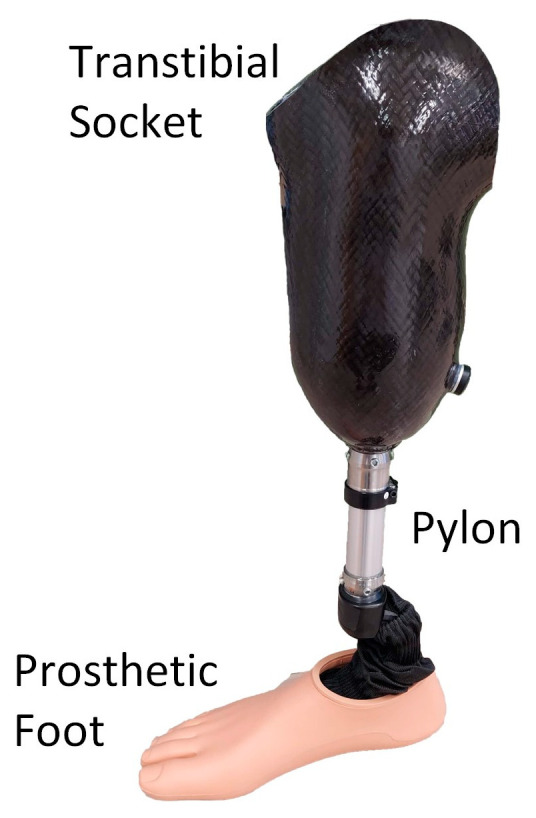
Conventional definitive transtibial prosthesis including a socket, pylon, and prosthetic foot.

Two primary socket designs are widely used in clinical practice: Total Surface Bearing (TSB) and Specific Surface Bearing (SSB), with Patellar Tendon Bearing (PTB) being particularly common. These designs involve the selective addition or removal of material to create relief areas and redistribute the load on the residual limb [15]. The fabrication of the prosthetic socket begins with examining the stump and creating a negative mold by shaping plaster directly on the limb [16,17]. The final prosthetic socket, typically made from composite materials such as carbon fiber, has a thickness of 3 to 6 mm, providing a sufficient stiffness-to-weight ratio (Figure 1) [18,19].

Prosthetic liners are crucial for bridging fit discrepancies between the stiff socket and the residual limb, impacting the prosthesis’s comfort and functionality [20]. The choice of liner is critical for stress reduction, volume adjustment, heat management, and the suppression of bacterial growth [20,21,22]. Common elastomeric liners, like silicone and gel, resist compression for a secure fit but may become uncomfortable with limb volume changes [23]. Closed-cell foam liners offer a more affordable option with better durability and lower maintenance, though with poorer mechanical properties [20,24]. Selection relies on prosthetists’ experience and supplier information [25]. Despite the trend towards non-compressible elastomeric liners, the need for cost-effective, customizable liners persists, requiring comparative studies and material testing [26]. Recent research suggests that innovative 3D-printed cellular metamaterial liners could overcome current limitations [27,28,29].

Recent advances in integrated design and computer-aided technologies (CAD/CAM/CAE), combined with 3D printing, present promising solutions for enhancing prostheses fit, safety, and performance [30]. The proposed digital manufacturing process starts with capturing the stump’s geometry via 3D scanning, Computed Tomography (CT), Magnetic Resonance Imaging (MRI), or similar methods, followed by virtual rectification, prosthesis design, and 3D printing [31,32,33]. Despite its potential, clinical use is limited by the lack of effective digital rectification methods to integrate these technologies seamlessly [34]. The Finite Element Method (FEM) can then identify regions of higher contact pressure for socket and liner modifications. The FEM has been shown to reduce prosthetic development costs by minimizing limb–socket fit iterations, therefore reducing the number of visits to prosthetists [35]. This allows for an initial design evaluation and an efficient biomechanical assessment of limb–prosthesis systems, enhancing fit, comfort, stability, and safety without physical prototypes [36,37,38]. 

Numerous specific lower-limb Finite Element (FE) models have been developed to analyze the donning of the socket, stance phase, or gait cycle [39]. While patient-specific lower-limb FE models provide insights into socket–liner systems, their applicability to diverse cases is limited due to unique patient geometry. To gain broader insights, several generic lower-limb FE models with simplified geometry have been proposed for the objective evaluation and development of socket–liner systems for general patient groups [40]. Statistical Shape Modeling (SSM) can be used to obtain an average geometry, balancing computational costs and accuracy [41]. However, the majority of generic lower-limb models are outdated or overly simplified, reducing their effectiveness with modern computer-aided technologies [42,43,44]. A recent study introduced a more advanced model using nonlinear material models and realistic boundary conditions, providing more accurate biomechanical insights [40]. 

Combining advancements in FE models with 3D printing technologies bridges the gap between accurate biomechanical insights and practical, customizable fabrication [31]. Stereolithography (SL), Selective Laser Sintering (SLS), Powder Bed Fusion, and Fused Filament Fabrication (FFF) have been used for lower-limb prosthetic socket fabrication [45,46,47,48]. Despite variations in cost and performance, these techniques share the principle of layer-by-layer construction. Advances in additive manufacturing and printable materials enable 3D-printed parts to be used as final products [31,49,50]. Additive manufacturing can improve conventional sockets by incorporating compliant areas that reduce stress concentration, enhancing fit and comfort [51]. A recent study demonstrated the safety and functionality of a low-cost 3D-printed transtibial prosthesis made from Polylactic Acid (PLA) filaments using FFF in Sierra Leone [52]. The development of 3D printing technology shows great potential in manufacturing lower-limb prosthetic sockets and custom prosthetic liners to enhance comfort, fit, and rehabilitation outcomes [20]. Three-dimensional printing offers benefits unattainable with traditional manufacturing, such as design freedom, mass customization, waste minimization, complex structure manufacturing, and rapid prototyping. Furthermore, custom liners can provide a personalized anatomical shape, spatially varying stiffness, and stress–strain responses. FEM-informed liner design has already been explored to automate and enhance the design process [27,28,29].

Further, 3D-printed flexible metamaterial cellular structures have also shown significant promise in biomechanics, since using such structures is particularly valuable for improving interactions between soft tissue and products/devices [53,54,55,56]. By studying the mechanical response of the soft tissue of the residual limb (skin, subcutaneous tissue, muscles, etc.), which exhibits low stiffness at small strains and increases substantially with higher strains, a prosthetic liner should, based on bioinspiration, exhibit inverse mechanical behavior. This entails maintaining stiffness at low contact pressures and deforming gradually at higher contact pressures to minimize stress concentrations. Silicone liners typically exhibit an exponential mechanical behavior, characterized by low stiffness at small strains and a significant increase in stiffness with higher strains. While this behavior closely mirrors that of soft tissue, it also means that silicone liners deform easily under lower strains, effectively reducing low contact pressures, but hereby also unnecessarily increasing liner deformation and hence losing prosthesis stability. As the strain increases, the liner’s capacity to deform diminishes, limiting its ability to further reduce contact pressure under higher stresses. These higher stresses are critical for soft tissue, where the risk of discomfort and damage is greatest.

It has been shown that 3D-printed flexible metamaterial cellular structures can be designed to deform at specific pressure thresholds, thereby minimizing stress concentrations [57]. By adjusting factors such as the base material, cell morphology (open or closed), topology, geometry, and relative density (the ratio between cell size and the density of the base material), these cellular structures can be optimized using bioinspired principles for significant improvements in numerous applications [58,59,60,61]. Hence, bioinspired 3D-printed metamaterial cellular structures show great potential in prosthetic liners, since they can reduce high and non-uniform contact pressures, enhancing patient comfort. Additionally, they can maintain the stability of the entire prosthesis by allowing for the controlled deformation of the liner only in regions with high contact pressures. Hence, these architected cellular metamaterials bridge the gap between conventional materials, offering the flexibility to design prostheses from various base materials to achieve specific bioinspired mechanical properties.

Despite the significant advancements in prosthetic socket and liner design, several shortcomings remain in current approaches. Traditional manufacturing techniques are labor-intensive, reliant on prosthetist expertise, and prone to inefficiencies and inaccuracies. The non-compressible nature of commonly used elastomeric liners may lead to discomfort. Furthermore, while 3D printing and FEM have revolutionized the design and testing phases, the development, analysis, and manufacturing of novel liners using 3D-printed flexible metamaterial cellular structures remain mostly unexplored.

Therefore, this paper aims to evaluate various unit cells of metamaterial structures and, using a previously developed and validated FEM model of a limb–liner–socket system with realistic boundary conditions, assess different stiffness levels of 3D-printed metamaterial structures, utilizing the optimal unit cell. This paper seeks to tailor lower-limb prosthetic liners to achieve lower and more uniform contact pressures between the residual limb and the prosthesis while ensuring the stability of the prosthesis, surpassing the performance of existing liners. 

The remainder of this paper is organized as follows: Section 2 presents the used materials and methods, with a focus on bioinspired design, metamaterial unit cell identification and analysis, cellular metamaterial sample mechanical testing, and the development of the FE model of the limb–liner–socket system. Section 3 details the results obtained from the FEM analysis. Section 4 discusses the implications of the findings in the context of different liner performances in terms of contact pressure and deformation, and improving prosthetic liner design in general. Finally, Section 5 concludes this paper, summarizing the key contributions and suggesting directions for future research.

## 2. Materials and Methods

**Figure 2:**
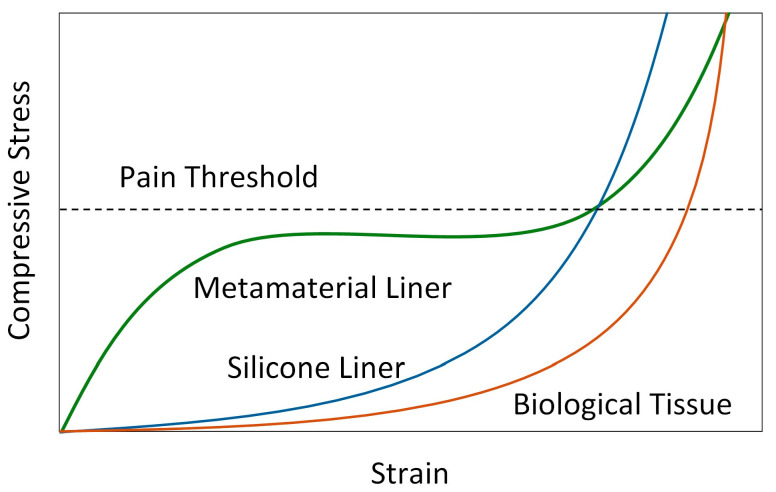
Stress–strain chart illustrating the response of the cellular liner, soft tissue, and silicone liner, with the pain threshold level indicated.

The proposed bioinspired design in this paper leverages metamaterial cellular structures in prosthetic liners to reduce contact pressure and enhance user comfort by mirroring the mechanical behavior of soft tissue, ensuring that high contact pressures are effectively reduced, and that controlled liner deformation does not compromise prosthesis stability. The cellular structure maintains a degree of stiffness until it approaches the pain pressure threshold, primarily allowing for soft tissue deformation to ensure stability. As this threshold is approached, the liner begins to deform, redistributing pressure over a larger area to minimize stress concentration and prevent discomfort. The deformation mechanism of the cellular structure includes a plateau region where deformation occurs under nearly constant stress, facilitating pressure redistribution. Figure 2 illustrates a schematic of the liner, biological soft tissue, and silicone liner response on a stress–strain diagram, including the pain threshold. The adaptability in material properties offered by metamaterial cellular structures makes achieving these design goals feasible.

### 2.1. Metamaterial Unit Cell Identification

To identify the optimal unit cell, various cell designs were evaluated using nTopology (nTopology Inc., New York, USA). The main objective was to find the cell design that exhibited the most isotropic response. An isotropic response is essential for prosthetic liners due to complex boundary conditions, which involve forces acting in multiple directions during use. Particular attention was given to Triply Periodic Minimal Surface (TPMS) structures, which are mathematical constructs that define a surface using three periodic functions in three dimensions [62]. These TPMS structures are widely used in Materials Science for the development of metamaterials with unique properties [63,64]. This study focused on several common TPMS structures, listed below [65]:

1. The gyroid structure;
2. The Schwarz structure;
3. The Neovius structure;
4. The diamond structure.

Each of these structures was analyzed to assess its potential for providing an isotropic mechanical response under the varying forces encountered in prosthetic applications.

Along with the TPMS structures, various commonly used beam unit cells were also analyzed. These unit cells consist of a lattice of interconnected beams, forming a repeating pattern throughout the structure. The geometry and orientation of the beams within the unit cell significantly affect the mechanical properties of the structure, including its stiffness, strength, and ductility [65]. The beam unit cells examined in this study included the following:

1. Simple cubic unit cell;
2. Octet unit cell;
3. Beam diamond unit cell;
4. Re-entrant unit cell.

**Figure 3:**
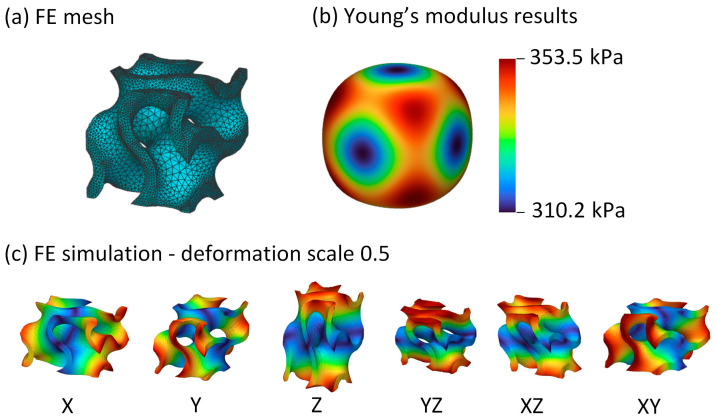
FEA of a unit cell performed in nTopology: (**a**) the meshed unit cell, (**b**) a spatial representation of the stiffness matrix, and (**c**) a representation of the deformation in all six directions.

In the initial stage, the geometric configuration of the unit cell was designed to include a wall or beam thickness of 1 mm within a cubic volume measuring 6 mm × 6 mm × 6 mm, corresponding to the liner’s thickness. Subsequently, the unit cells underwent meshing, and a Finite Element Analysis (FEA) was automatically conducted using nTopology software (version 4.26.4) as illustrated in Figure 3. The edge length of the FE mesh was 0.5 mm, with a growth rate of 1.2 mm, yielding a total of 49,219 elements and 81,612 nodes for a gyroid unit cell.

Six distinct loading conditions were investigated: three along the normal direction and three along the shear direction, resulting in the formulation of a 6 × 6 stiffness matrix (Equations (1) and (2)). This matrix is intended for the homogenization of the cellular material model in numerical simulations. In the isotropic formulation of Hooke’s law, the stiffness matrices are characterized by only two independent variables, namely, the elastic constants, in contrast to the 21 elastic constants present in the general anisotropic case [66]. These elastic constants are typically expressed in terms of Young’s modulus (E) and Poisson’s ratio (ν), although they can alternatively be defined using the bulk modulus (K) and/or shear modulus (G). The equations are able to derive G and K from E and ν, and vice versa, for isotropic materials [66].

Cauchy extended Hooke’s law to three-dimensional elastic bodies within the framework of anisotropic materials, establishing that the six stress components exhibit linear correlations with the six strain components (Equation (3)). This stress–strain relationship can be formulated in matrix notation, where the six stress and strain components are organized as column vectors:

(1) $$\left[\sigma \right]=\left[C\right]\cdot \left[\varepsilon \right],\;\;\left[\begin{array}{c} \sigma_{xx}\\ \sigma_{yy}\\ \begin{array}{c} \sigma_{zz}\\ \sigma_{yz}\\ \begin{array}{c} \sigma_{xz}\\ \sigma_{xy} \end{array} \end{array} \end{array}\right]=\left[\begin{array}{ccc} \begin{array}{ccc} C_{11} & C_{12} & C_{13}\\ C_{21} & C_{22} & C_{23}\\ C_{31} & C_{32} & C_{33} \end{array} & \begin{array}{c} C_{14}\\ C_{24}\\ C_{34} \end{array} & \begin{array}{c} \begin{array}{cc} C_{15} & C_{16} \end{array}\\ \begin{array}{cc} C_{25} & C_{26} \end{array}\\ \begin{array}{cc} C_{35} & C_{36} \end{array} \end{array}\\ \begin{array}{ccc} C_{41} & C_{42} & C_{43} \end{array} & C_{44} & \begin{array}{cc} C_{45} & C_{46} \end{array}\\ \begin{array}{c} \begin{array}{ccc} C_{51} & C_{52} & C_{53} \end{array}\\ \begin{array}{ccc} C_{61} & C_{62} & C_{63} \end{array} \end{array} & \begin{array}{c} C_{54}\\ C_{64} \end{array} & \begin{array}{c} \begin{array}{cc} C_{55} & C_{56} \end{array}\\ \begin{array}{cc} C_{65} & C_{66} \end{array} \end{array} \end{array}\right]\left[\begin{array}{c} \varepsilon_{xx}\\ \varepsilon_{yy}\\ \begin{array}{c} \varepsilon_{zz}\\ \varepsilon_{yz}\\ \begin{array}{c} \varepsilon_{xz}\\ \varepsilon_{xy} \end{array} \end{array} \end{array}\right]$$

(2) $$\left[\varepsilon \right]=\left[S\right]\cdot \left[\sigma \right],\;\;\left[\begin{array}{c} \varepsilon_{xx}\\ \varepsilon_{yy}\\ \begin{array}{c} \varepsilon_{zz}\\ \varepsilon_{yz}\\ \begin{array}{c} \varepsilon_{xz}\\ \varepsilon_{xy} \end{array} \end{array} \end{array}\right]=\left[\begin{array}{ccc} \begin{array}{ccc} S_{11} & S_{12} & S_{13}\\ S_{21} & S_{22} & S_{23}\\ S_{31} & S_{32} & S_{33} \end{array} & \begin{array}{c} S_{14}\\ S_{24}\\ S_{34} \end{array} & \begin{array}{c} \begin{array}{cc} S_{15} & S_{16} \end{array}\\ \begin{array}{cc} S_{25} & S_{26} \end{array}\\ \begin{array}{cc} S_{35} & S_{36} \end{array} \end{array}\\ \begin{array}{ccc} S_{41} & S_{42} & S_{43} \end{array} & S_{44} & \begin{array}{cc} S_{45} & S_{46} \end{array}\\ \begin{array}{c} \begin{array}{ccc} S_{51} & S_{52} & S_{53} \end{array}\\ \begin{array}{ccc} S_{61} & S_{62} & S_{63} \end{array} \end{array} & \begin{array}{c} S_{54}\\ S_{64} \end{array} & \begin{array}{c} \begin{array}{cc} S_{55} & S_{56} \end{array}\\ \begin{array}{cc} S_{65} & S_{66} \end{array} \end{array} \end{array}\right]\left[\begin{array}{c} \sigma_{xx}\\ \sigma_{yy}\\ \begin{array}{c} \sigma_{zz}\\ \sigma_{yz}\\ \begin{array}{c} \sigma_{xz}\\ \sigma_{xy} \end{array} \end{array} \end{array}\right]$$

(3) $$S=C^{-1}$$

where

***σ*** [Pa]: −6 × 1 stress vector
***ε*** [-]: −6 × 1 strain vector
**C** [Pa]: −6 × 6 stiffness matrix
**S** [Pa^−1^]: −6 × 6 compliance matrix

The diagonal terms represent the stiffness coefficients for normal stresses in the x, y, and z directions, while the non-diagonal terms relate to the stiffness coefficients for the shear stresses in the xy, xz, and yz planes. Ordinarily, the stiffness matrix comprises 36 components. However, in the case of conservative materials with a SED function, the stiffness and compliance matrices are symmetric. Hence, only 21 stiffness components in Hooke’s law are independent.

This study investigates the modulus of elasticity in three-dimensional space as a measure of the stiffness of cellular units in different directions. The material used in the simulation was thermoplastic polyurethane (TPU), which has a modulus of elasticity of 2410 MPa and a Poisson’s ratio of 0.3897. The unit cells analyzed were evaluated based on the difference between the maximum and minimum values of the modulus of elasticity, which is independent of the material used and instead depends on the topological, morphological, and geometrical characteristics of the unit cells [65].

**Figure 4:**
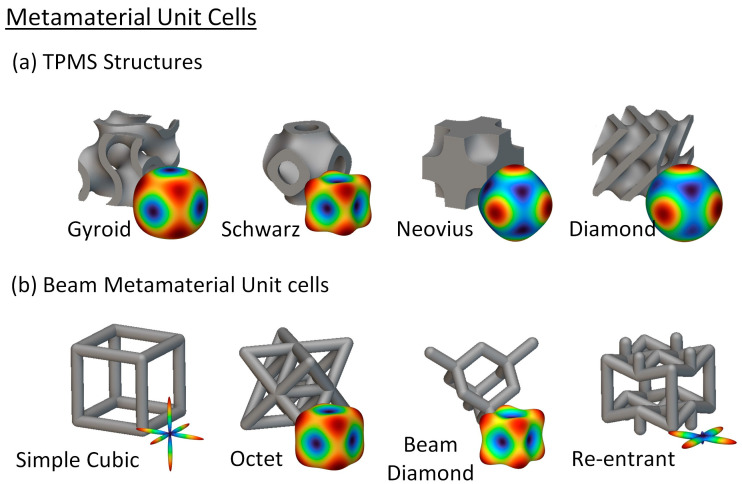
Unit cell structures used in the FEA along with the spatial representation of the stiffness matrix: (**a**) TPMS structures and (**b**) beam metamaterial structures. In the stiffness matrix, red represents higher stiffness, while blue indicates lower stiffness.

Figure 4 presents the analyzed unit cells alongside a visual representation of the stiffness matrix. In this representation, red indicates areas of higher stiffness, while blue indicates areas of lower stiffness. This analysis reveals how different unit cell geometries affect the overall mechanical behavior of metamaterial structures under various loading conditions.

The orientation of beams within the beam metamaterial unit cells plays a critical role in determining the stiffness matrix, as illustrated in Figure 4. In the simple cubic unit cell, the parallel arrangement of beams results in a highly directional stiffness response, with increased stiffness confined to the direction of the beams. This characteristic is also observed in the re-entrant structure. In contrast, the octet and diamond unit cells exhibit a more uniform stiffness distribution, attributable to their more interwoven beam orientations.

The findings presented in Table 1 indicate that TPMS structures demonstrate a more consistent response in comparison to beam structures. Particularly, the gyroid and Neovius TPMS unit cells show the most advantageous outcomes, with modulus ranges of 12.2% and 11.52%, respectively. Given the minimal disparity between these unit cells, the gyroid structure was chosen due to its widespread adoption and ease of 3D printing, as gyroid patterns are commonly integrated into commercial slicers as infill patterns.

### 2.2. Multilinear Elastic Material Model

Upon initial examination of the metamaterial unit cells, it is observed that the gyroid structure exhibits nearly isotropic material properties. The structure’s compatibility with cost-effective FFF printing methods positions it as a promising alternative for prosthetic liner applications. However, the thickness of individual cells aligns with that of the liner, necessitating caution when using a homogenized material model derived directly from the stiffness matrix obtained in simulations, as this can yield unreliable numerical outcomes. To apply such an approach effectively in FEA, a thickness approximately ten times that of a single cell is recommended to mitigate the influence of cell walls. Additionally, the exclusion of the cellular structure’s walls from unit cell simulations further contributes to potential inaccuracies.

Based on the aforementioned considerations, the mechanical behavior of the 3D-printed gyroid structure, fabricated using flexible TPU filaments, was characterized using compression test data from our previous research [57]. Samples measuring 30 × 30 mm were printed using an FFF 3D printer and TPU material sourced from a local supplier, AzureFilm (AzureFilm d.o.o., Sežana, Slovenia), marketed under the brand name Flexible 98A. This chosen material is non-corrosive, minimally irritating to the skin, and has a melting point exceeding 100 °C, ensuring its safety for applications involving contact between the product and biological tissues. Furthermore, TPU offers a balance between flexibility and durability, which is critical for prosthetic liners that must conform to the residual limb’s shape while enduring repetitive loads during gait. Gyroid samples with infill densities of 6%, 10%, and 14% were investigated based on data extracted from prior uniaxial compression tests. Infill density represents the ratio of the internal volume occupied by solid material to the total volume of the structure. The chosen densities were selected based on our previous analyses, as they yield distinct responses corresponding to soft, medium, and hard structural characteristics [57]. Henceforth, the gyroid structures with 6%, 10%, and 14% density will be referred to as soft, medium, and hard, respectively.

To achieve a numerically stable representation of the mechanical response of 3D-printed metamaterial structures, a multilinear elastic material model (MELAS) was employed. The use of the MELAS approximates the mechanical behavior of the metamaterial structure without directly incorporating its intrinsic geometry, thereby promoting numerical stability. In contrast, using a hyperelastic material model to fit the response curves would introduce greater numerical instability. Consequently, the stress–strain curves of the structures were manually fitted using three distinct slopes per material.

**Figure 5:**
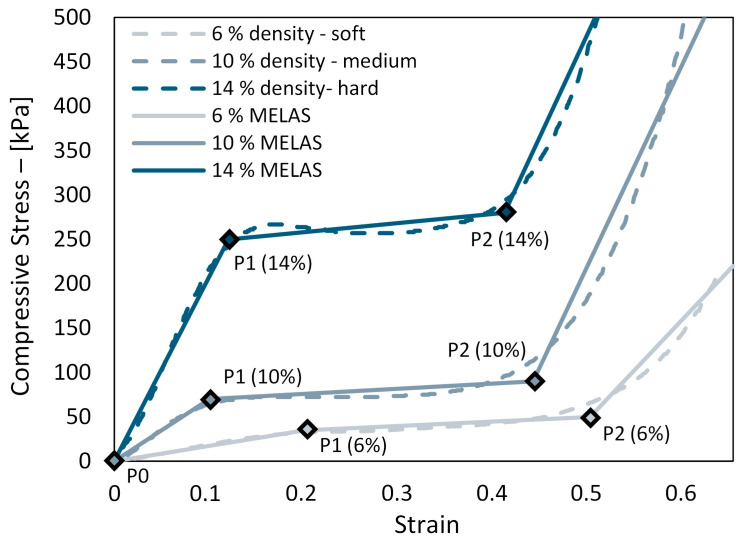
Results of the uniaxial compression test with 6% (soft), 10% (medium), and 14% (hard) gyroid infill patterns. Solid lines represent the MELAS models used in numerical simulations to capture the hyperelastic behavior of the cellular structures. Diamond markers indicate the start and finish of the plateau regions for each structure.

The first slope represents the initial stiffness of the structure, followed by a plateau region corresponding to the deformation of the cell walls, and finally, the last steep slope denotes the densification of the cells. A noteworthy difference in stiffness between the soft, medium, and hard structure was observed, suggesting the potential for designing cellular metamaterial structures with tailored mechanical properties. The results are illustrated in the stress–strain graph in Figure 5. The solid lines indicate the MELAS material model, which approximates the hyperelastic response of the tested samples. The original responses of the metamaterial samples are shown with dotted lines. Furthermore, the stress–strain points used to define the MELAS material model for cellular metamaterial structures with varying densities are provided in Table 2.

### 2.3. Numerical Analysis

**Figure 6:**
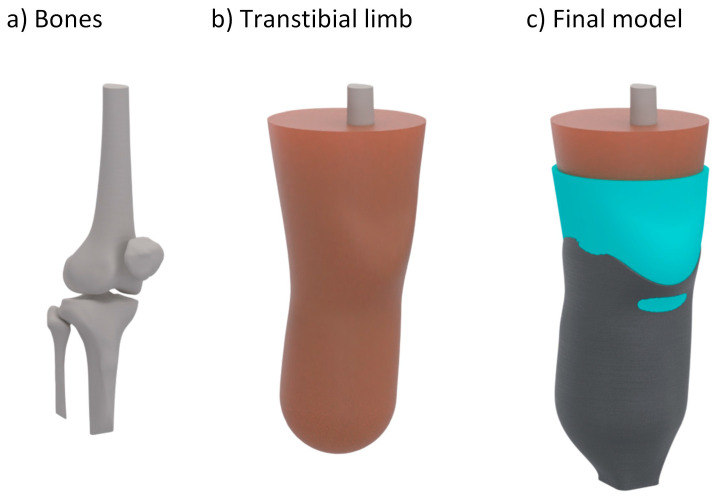
Geometry of the generic transtibial limb–prosthesis model: (**a**) bones, including the patella, tibia, fibula, and femur; (**b**) the soft tissue of the residual limb; and (**c**) the final model, including the transtibial limb, prosthetic liner, and socket.

The newly developed bioinspired metamaterial liners were assessed using a previously validated average-size generic transtibial model under real-life conditions simulated in a virtual environment. This model encompasses bulk soft tissue and bones, including the tibia, femur, fibula, and patella, scaled to reflect the dimensions of an average adult male [40]. The bone geometry and the generic transtibial limb model are illustrated in Figure 6a and Figure 6b, respectively.

**Figure 7:**
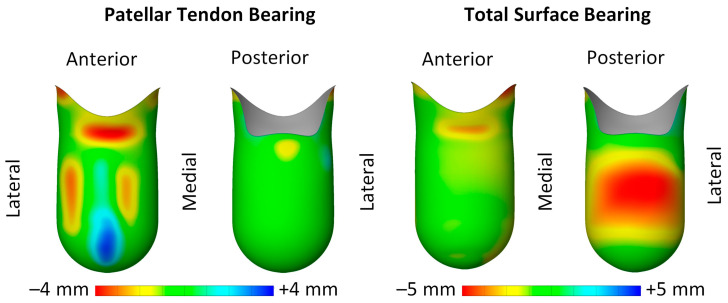
Color map illustrating rectification of PTB and TSB socket, where red denotes depressed areas and blue indicates domed areas.

The socket had a thickness of 3 mm, aligning with the typical range of 3 mm to 6 mm [67]. Figure 7c depicts the final model, illustrating the initial overlap of the socket. This overlap was addressed in the initial phase of the numerical analysis, which simulated the donning process of the prosthesis.

A 6 mm constant thickness liner was incorporated into the model by extending the outer surface of the soft tissue, thereby conforming to the residual limb’s shape in a manner analogous to the application of a silicone liner. Following this, the transtibial socket was rectified according to standard Patellar Tendon Bearing (PTB) and Total Surface Bearing (TSB) rectification guidelines to redistribute pressure from sensitive areas to pressure-tolerant regions, hence improving comfort (Figure 7).

Due to the complex, patient-specific biological structure of soft tissue, defining a material model applicable to the entire population presents significant challenges. However, several key properties must be addressed to accurately mimic the response of soft tissue. Firstly, the bulk soft tissue exhibits near incompressibility, necessitating a modeling approach that reflects this characteristic. Secondly, the strain–stress response of soft tissue under compression is nonlinear; at low strain, the tissue offers minimal resistance, whereas at higher strain levels, it becomes increasingly stiffer. To capture this nonlinear response, a hyperelastic material model was employed in the simulation to define the soft tissue [68]. 

Additionally, a hyperelastic material was utilized to model the commonly used silicone liner. A multilinear elastic material model was employed for the newly developed metamaterial cellular liners, based on the findings from the uniaxial compression tests described in the previous subsection. For the socket, a 3D-printed PLA material was utilized, which had been previously validated through uniaxial testing in earlier research [69]. The material models implemented in the simulations, including their parameters and references, are comprehensively detailed in Table 3.

Compared to soft tissue, the stiffness of bones is significantly higher, resulting in negligible deformations within the model. Consequently, bones were modeled as rigid cavities bonded to the soft tissue, thereby reducing the computational time of the numerical simulation. A rough contact was established at the residual limb–liner interface, permitting separation in the normal direction while restricting sliding. At the liner–socket interface, a coefficient of friction of 0.5 was employed, based on a previous study [43].

**Figure 8:**
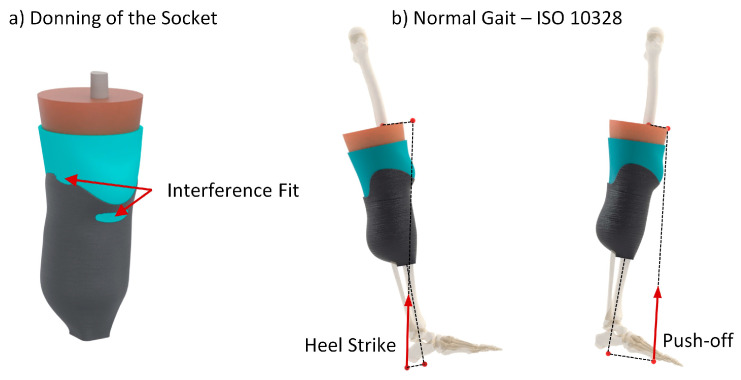
Loading conditions applied in the numerical analysis, comprising (**a**) socket donning and (**b**) normal gait, adhering to ISO 10328 guidelines.

The initial loading condition evaluated in the simulation was the socket donning phase. Prior studies have demonstrated that the stress occurring after the socket is donned on the residual limb affects subsequent steps, necessitating its inclusion in the simulation [70,71]. The donning process was simulated by resolving the initial overlap between the residual limb and the liner (Figure 8a). The subsequent steps included two critical moments in the gait cycle: heel strike and push-off (Figure 8b). The position and magnitude of the load vector were derived from the ISO 10328 standard [72] for the structural testing of lower-limb prostheses, specifically for the P5 loading level and settling test force, reflecting the loads experienced during normal gait for individuals weighing up to 100 kg.

## 3. Results

**Figure 9:**
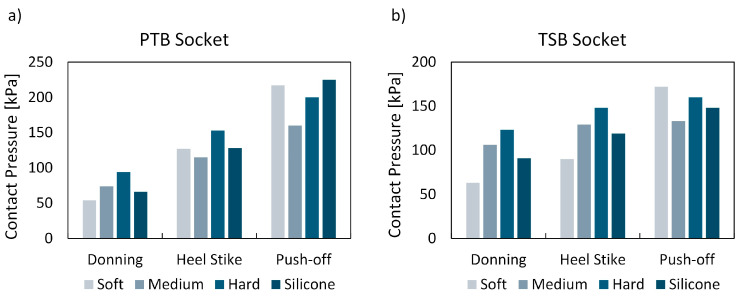
Maximum contact pressure during donning, heel strike, and push-off phases for all liner types, using (**a**) PTB socket and (**b**) TSB socket.

**Figure 10:**
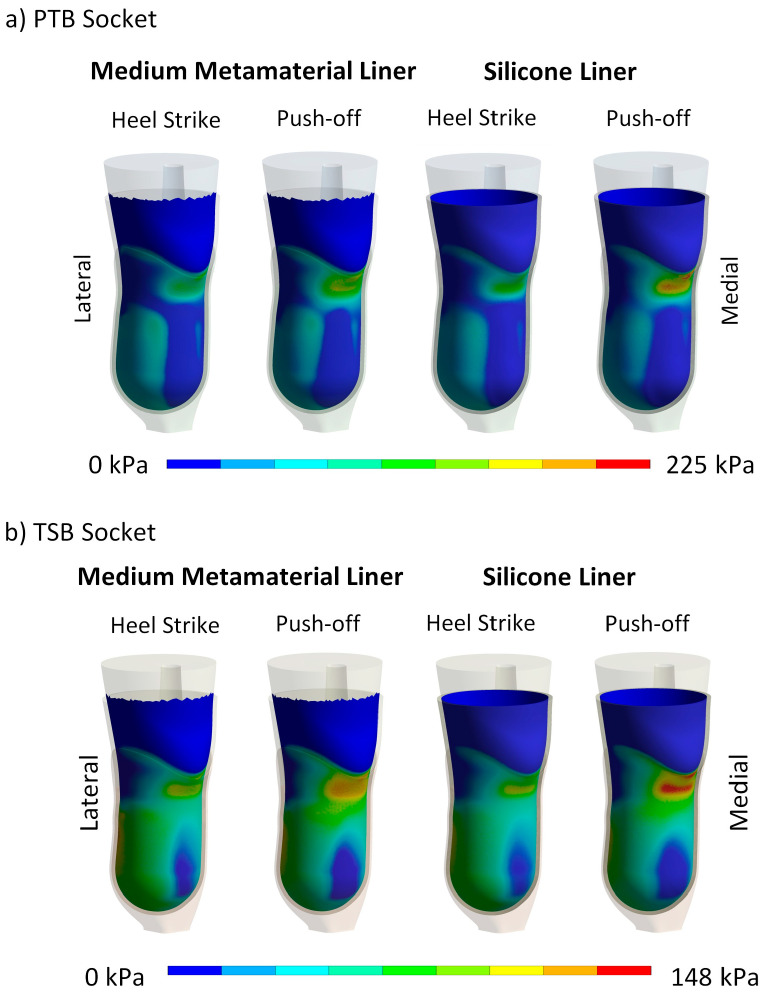
Comparison of contact pressure distribution between a medium metamaterial liner and a traditional silicone liner during the heel strike and push-off phases for (**a**) the PTB socket and (**b**) TSB socket.

The FEA was conducted to analyze and assess various liners concerning residual limb–liner contact pressure and the total deformation of a liner within a virtual environment. Evaluating the comfort of prosthetic liners involves two critical factors: peak pressure and pressure distribution. Given the use of a generic transtibial model, the results should be interpreted in relative terms, focusing on the differences between liners rather than on specific values. Figure 9 presents the peak contact pressure results for all liner types, loading conditions, and rectification methods. Additionally, Figure 10 compares the pressure distribution between the residual limb and the soft metamaterial liner and a conventional silicone liner. It is noteworthy that the pressure distribution was similar across all metamaterial liner densities. 

**Figure 11:**
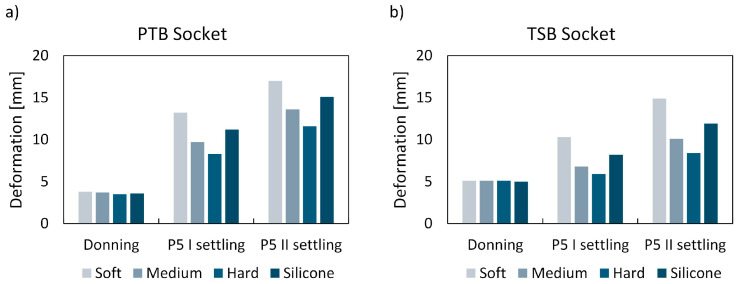
Maximum deformation during donning, heel strike, and push-off phases for all liner types, using (**a**) PTB socket and (**b**) TSB socket.

**Figure 12:**
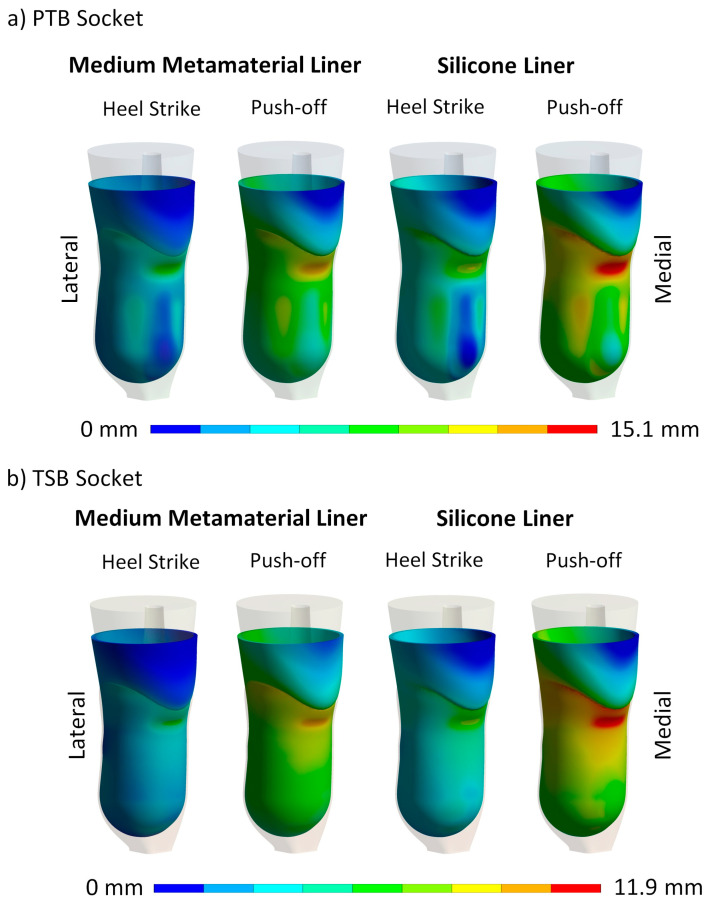
Comparison of deformation distribution between a medium metamaterial liner and a traditional silicone liner during the heel strike and push-off phases for (**a**) the PTB socket and (**b**) the TSB socket.

The stability of prostheses is a critical factor impacting user safety and confidence during use. To assess the liner’s contribution to prosthetic stability, the total deformation of the liner was assessed. This evaluation encompassed the combined translation and deformation of the liner. The maximum deformation values for all liner types, loading cases, and rectification methods are presented in Figure 11. Additionally, Figure 12 illustrates the deformation distribution, highlighting areas (in red) where the liner experiences the highest deformation. 

All numerical data regarding contact pressure and maximum deformation obtained through the FEA are compiled and presented in Table 4. The upper section of the table displays the results for the PTB socket, while the lower section presents the results for the TSB socket.

## 4. Discussion

The biomechanical interaction between prosthetic liners and residual limb tissues is crucial in determining the comfort, stability, and overall functionality of lower-limb prostheses. Understanding the complex mechanical behaviors of soft tissues, particularly their nonlinear stress–strain responses, is essential for designing prosthetic liners that effectively mitigate pressure-related issues. Soft tissues exhibit significant deformability under low loads, with stiffness increasing exponentially under higher stresses. This characteristic allows for effective pressure redistribution, thereby reducing the risk of tissue damage due to stress concentration. However, challenges arise in areas with minimal soft tissue thickness, such as the tibial crest or fibular head, where exceeding the tissue’s deformation capacity can lead to discomfort and potential injury.

Conventional prosthetic liners, predominantly made of elastomers, exhibit high elasticity but lack compressibility, limiting their ability to adapt to varying pressure conditions across the residual limb. This inherent material property often results in non-uniform contact pressures, with localized peaks that can induce discomfort, pain, and even musculoskeletal disorders. Under a thorough understanding of the prosthesis–liner–stump biomechanical system using the developed FE model, the prosthetic liner should, based on bioinspiration, exhibit inverse mechanical behavior to soft tissue. This entails maintaining stiffness at low contact pressures and deforming gradually at higher contact pressures to minimize contact pressure concentrations and maintain stability. This stress–strain behavior allows the metamaterial to reduce contact pressure more effectively where it is most needed—under high-stress conditions. By staying stiff at low pressures and becoming more compliant at higher pressures, the metamaterial structure offers a significant advantage in protecting soft tissue, enhancing comfort, and ensuring better fit and stability in prosthetic liners.

In this context, TPU was selected due to its unique combination of properties: it is non-corrosive, minimally irritating to the skin, and has a melting point exceeding 100 °C, ensuring its safety for applications involving contact with biological tissues. Furthermore, TPU offers a balance between flexibility and durability, which is critical for prosthetic liners that must conform to the residual limb’s shape while enduring repetitive loads during gait. Compared to other materials commonly used in prosthetic liners, such as silicone and thermoplastic elastomers (TPEs), TPU provides significant advantages. Silicone, while highly biocompatible and resistant to wear, is heavier and more challenging to process using additive manufacturing techniques. TPEs offer excellent elasticity and ease of processing, but may lack the mechanical strength and thermal stability offered by TPU. This analysis underscores the suitability of TPU for our application, particularly in the context of 3D printing, where its processability and material properties provide critical benefits. However, TPU’s lower resistance to wear compared to silicone is acknowledged as a potential limitation, particularly for long-term use, which will be a focus of our future investigations. Furthermore, TPU’s recyclability presents a significant advantage. The material and the additive manufacturing process used to produce the liners allow for the recycling and reprocessing of worn or damaged liners, which not only extends the product’s lifecycle but also contributes to reducing environmental impact. This characteristic aligns with the broader goals of sustainability in prosthetic device design, offering an avenue for future research into the development of more durable and eco-friendly prosthetic liners. 

The contact pressure distribution is significantly affected by the rectification method used. For example, in the PTB socket, the highest contact pressure is detected at the patellar tendon area, displaying a more concentrated pattern. Conversely, the TSB socket shows a peak pressure at the posterior region, with a more even distribution across the residual limb. These pressure distribution patterns in the PTB and TSB sockets align with observations from other studies in the field. The findings indicate that the numerical model successfully produced pressure values aligning with those documented in earlier studies, assuming comparable definitions were applied. A thorough verification and validation of the numerical model was detailed in our previous publication [40].

### 4.1. Unit Cell Selection

Identifying the optimal unit cell is critical for achieving the desired mechanical properties in cellular prosthetic liners, mainly aiming for a high degree of isotropy. Isotropic responses are essential due to the complex multidirectional forces these liners encounter during use. Our analysis revealed that TPMS structures generally exhibit more consistent isotropic behavior compared to beam structures. Among the TPMS structures studied, the gyroid and Neovius unit cells stood out, with modulus ranges of 12.2% and 11.52%, respectively. These structures demonstrate favorable outcomes in terms of the minimal variation in Young’s modulus, indicating uniform stiffness across different directions. In contrast, the beam structures, such as simple cubic, octet, beam diamond, and re-entrant structures, displayed significantly higher variations in stiffness. This variability suggests more anisotropic behavior, potentially leading to inconsistent performance and discomfort for users. 

The gyroid structure was selected as the optimal choice due to having several advantages: a widespread adoption, an ease of 3D printing, and minimal disparity between maximum and minimum Young’s modulus values. Gyroid patterns are commonly integrated into commercial slicers as infill patterns, simplifying the manufacturing process. Moreover, its smooth, continuous minimal surface and efficient load distribution properties make it ideal for applications requiring lightweight yet strong materials. By focusing on the gyroid structure, this study ensures that prosthetic liners can perform consistently under diverse loading conditions. This decision is supported by the structure’s inherent isotropy, which is crucial for addressing the complex mechanical demands encountered in prosthetic applications.

### 4.2. Maximum Contact Pressure and Maximum Liner Deformation

Contact pressure is a crucial metric for assessing the comfort of lower-limb prostheses [33]. Numerical simulations were conducted to evaluate the maximum contact pressure between the residual limb and cellular liners with different infill densities, and these results were compared to those obtained with silicone liners (Figure 9 and Figure 11 and Table 4). The results in the continuation are discussed in relative comparison between different liner materials and are hence provided in percentages of reduction or increase. During the donning phase, a soft metamaterial liner demonstrated an 18% and 31% reduction in peak contact pressure compared to the silicone liner for PTB and TSB rectification, respectively. Conversely, a medium–dense liner showed an increase in peak contact pressure of 12% (PTB) and 17% (TSB), while a hard liner showed an even higher increase in contact pressure of 42% (PTB) and 35% (TSB). When analyzing the results of maximum deformation for the donning load case, it is clear that all liner materials deform almost equally, with the highest difference being 0.3 mm (8%) between the soft and hard metamaterial liners for the PTB rectification method. The most significant difference between the cellular metamaterial and silicone liner is observed with the soft liner, specifically, 0.2 mm (5%) for the PTB socket. Since the donning phase is not a part of an active gait phase, and hence stability, the liner deformation does not play a crucial role. Hereby, individuals with a more sedentary lifestyle would most likely prefer the soft cellular liner (6% infill density), since it results in significantly lower peak contact pressures. 

However, when considering the active gait phase, such as heel strike and push-off, higher stress on the socket–liner–stump biomechanical system results in overall higher contact pressures. In this regard, the higher stiffness of the cellular liners shows more favorable results. For the heel strike, the medium liner showed a 10% reduction in peak contact pressure, while simultaneously reducing the maximum liner deformation by 13% compared to the silicone liner for PTB rectification. TSB rectification showed an 8% increase in peak contact pressure, with a significantly lower cellular liner deformation of 17% compared to the silicone liner. The hard cellular liner resulted in a 20% increase in peak contact pressure and a 26% reduction in liner deformation compared to the silicone liner for PTB rectification, and a 24% increase in peak contact pressure and a 28% reduction in liner deformation for TSB rectification. The soft cellular liner follows the same trend, showing a reduction in contact pressure of 1%, but with an increased cellular liner deformation of 18% compared to the silicone liner for PTB rectification, and a 24% reduction in peak contact pressure and a 26% increase in cellular liner deformation compared to the silicone liner for TSB rectification.

For the push-off phase of the gait cycle, stiffer (medium and hard) cellular liners showed more favorable results in terms of peak contact pressure and deformation. A medium cellular liner showed a 29% reduction in contact pressure and 10% less liner deformation compared to a silicone liner for PTB rectification, and a 10% reduction in peak contact pressure with a 15% reduction in peak liner deformation for TSB rectification. A hard cellular liner showed a slightly lower reduction in contact pressure of 11%, but also a significantly greater reduction in liner deformation of 23% compared to the silicone liner for PTB rectification. TSB rectification showed an 8% increase in peak contact pressure, accompanied by a significant 31% reduction in cellular liner deformation compared to the silicone liner. A soft cellular liner showed a 4% reduction in peak contact pressure and a 13% increase in liner deformation for PTB rectification. TSB rectification exhibited a 16% increase in contact pressure and a 25% increase in cellular liner deformation compared to the silicone liner.

The results suggest that while silicone liners provide good overall results in terms of contact pressure reduction, cellular liners exhibit greater potential for customization and performance optimization. However, the stiffness of the liner needs to be adjusted correctly to achieve the desired contact pressure reduction and liner deformation for maintaining stability. The cellular liner allows for fine-tuning and patient-specific customization in terms of activity levels. The soft cellular liner is well suited for individuals with a more sedentary lifestyle due to its significant reduction in peak contact pressure during the donning phase. The softer liner offers enhanced comfort and is less likely to cause pressure sores or discomfort for users who spend a lot of time sitting or are less active. The plateau level of the cellular structure is lower than that of medium and hard cellular liners. Hence, the significant reduction in the contact pressure during the donning phase compared to the silicone liner, especially for TSB rectification, indicates that the plateau has already been achieved. 

However, the results in terms of contact pressure for the active gait phases, such as heel strike and push-off for the soft cellular liner, are less favorable, since all cellular structures are already deformed, and the densification area has been reached. This, in conclusion, increases contact pressure for higher loading cases with extensive liner deformation. The lower deformation associated with a stiffer liner enhances stability and proprioception, critical for dynamic activities and maintaining balance. This increased stability is particularly important during the heel strike and push-off phases of gait, where control and feedback are essential for effective movement. Hence, for more active users, the stiffer cellular liners (medium and hard) show more favorable results in terms of peak contact pressure and liner deformation. The suggested medium cellular liner with 10% infill density would be suited for users with moderate activity levels, since this liner shows favorable reductions in peak contact pressure when compared to silicone liners, while also minimizing deformation, offering both comfort and support during active phases like heel strike and push-off. The hard cellular liner with 14% infill density would be suited for highly active users who require more stability and support during dynamic movements. Despite the peak contact pressure being almost the same as that of the silicone liner, the significant reduction in liner deformation ensures that the liner maintains its shape and provides consistent support during high-impact activities such as walking, running, cycling, climbing, etc. 

### 4.3. Contact Pressure Distribution and Liner Deformation

In addition to examining the maximum contact pressure, the pressure distribution at the limb–liner interface was analyzed, revealing significant differences between the PTB and TSB sockets under varying loads. Our focus was on the medium cellular liner with 10% infill density, which balanced comfort and stability most effectively (Figure 10 and Figure 12). Note that the pressure scales vary for each load step. The results indicate that contact pressure reduction in cellular liners stems from their structural deformation rather than material flow, as seen in silicone liners. Consequently, non-uniform and high contact pressures are mitigated by the deformation of the cellular structure rather than pressure redistribution due to silicone’s incompressibility. This is evident in the peak contact pressure distribution, where peak pressures are reduced through cellular structure deformation.

The contact pressure reduction mechanisms of cellular and silicone liners differ significantly. The cellular liner, inspired by bioinspired principles, employs a pre-engineered deformation plateau level that reduces pressure through deformation at the cellular level. In contrast, the silicone liner reduces contact pressure by material flow from high-pressure to low-pressure areas due to its incompressibility, which limits its pressure reduction capability. The cellular liner shows greater pressure reduction and more targeted deformation in high-stress areas, while the silicone liner exhibits extensive deformation due to material flow, highlighting the cellular liner’s superior performance in reducing contact pressure when choosing the appropriate cellular liner density.

Identifying a universally optimal structure for all prosthetic users is inherently difficult due to the inherent differences in physiological and psychological factors among individuals. Hence, the goal is to attain an enhanced equilibrium between comfort and stability based on individual patient needs and activity levels. This customization can be achieved by adjusting the infill density, which can tailor the mechanical properties of the liner to provide optimal comfort and performance.

Cellular metamaterial liners can be produced using cost-effective FFF printing technology. This significantly reduces the price compared to the rather expensive silicone liners. The affordability of cellular liners makes them accessible to a broader range of patients, including those who may require multiple liners. With the reduced cost and ease of production, patients could obtain several different liners for different activities. For example, a soft liner with a 6% infill density could be used for sedentary activities, while a hard liner with a 14% infill density could be reserved for sports and high-impact activities. This flexibility allows for patient-specific customization, enhancing comfort and performance across various scenarios.

For future work, the contact pressure and stability of prosthetic liners should be optimized using functionally graded cellular structures, tailoring stiffness to different areas of the residual limb. A stiffer cellular structure could support high-pressure areas, while a softer cellular structure would enhance comfort in sensitive regions by allowing for greater deformation. Additionally, the numerical model should also be expanded to account for variability in patient anatomical and topological features, such as residual limb shape and length. To further validate the findings of this study, future research should include the fabrication of 3D-printed samples of the evaluated metamaterial liners. This will allow for empirical testing and provide a practical assessment of the feasibility and performance of these structures in prosthetic applications. Manufacturing and testing these liners will help to correlate the numerical results with real-world performance and subjective comfort ratings. Additionally, studies on long-term durability, heat dissipation, and skin temperature regulation would be needed. 

## 5. Conclusions

This study examined the biomechanical performance of bioinspired, 3D-printed metamaterial cellular structures used as prosthetic liners to improve comfort and stability for lower-limb amputees. The numerical simulations demonstrated that metamaterials exhibited a favorable nonlinear deformation response, which contrasts with the behavior of soft tissue. This unique response effectively redistributed contact pressure over a larger area of the residual limb, alleviating stress concentrations and enhancing comfort during prosthesis use, which is essential for prolonged wear. Among the designs tested, the gyroid unit cell was identified as being particularly effective due to its isotropic properties, providing consistent stiffness in all directions. This characteristic ensures dependable performance under the multidirectional forces encountered during daily activities. Further evaluation of liners with varying stiffnesses and infill densities—specifically, medium and hard cellular liners with 10% and 14% infill densities—revealed significant reductions in peak contact pressures during dynamic gait phases such as heel strike and push-off, compared to traditional silicone liners. This improvement in pressure distribution was achieved without compromising stability, thanks to controlled deformation, which is crucial for maintaining balance and proprioception during physical activities. On the other hand, the soft liner with 6% infill density demonstrated superior biomechanical performance during the donning phase, suggesting enhanced usability for patients with more sedentary lifestyles. The ability to customize the stiffness of cellular metamaterial liners offers a significant advantage, allowing for the creation of personalized prosthetic liners that cater to individual needs. Whether the focus is on maximizing comfort and minimizing contact pressure or enhancing stability and proprioception, these liners present a versatile solution. Ultimately, the findings of this study underline the potential of cellular metamaterial liners to improve the quality of life for lower-limb amputees by providing tailored solutions that meet diverse biomechanical requirements. 

## Figures and Tables

**Table 1:** Stiffness range of analyzed unit cells expressed as percentages—variation between maximum and minimum Young’s modulus.

Metamaterial Unit Cell	Maximum Range of Young’s Modulus
TPMS Structures	
Gyroid	12.2%
Schwarz	45.8%
Neovius	11.5%
Diamond	17.7%
Beam Structures	
Simple Cubic	96.3%
Octet	33.7%
Beam Diamond	50.7%
Re-entrant	92.1%

**Table 2:** Stress–strain parameters for defining a MELAS material model that mimics a response of different cellular structures. P0–P3 denotes the points used to define the material model in the simulation.

	Soft (6% MELAS)		Medium (10% MELAS)		Hard (14% MELAS)	
Points	***σ*** [kPa]	***ε*** [-]	***σ*** [kPa]	***ε*** [-]	***σ*** [kPa]	***ε*** [-]
P0	0	0	0	0	0	0
P1	35	0.2	70	0.1	250	0.12
P2	50	0.5	90	0.44	280	0.41
P3	220	0.65	500	0.62	650	0.57

**Table 3:** Summary of the material models used in the simulations.

Component	Material Model	Reference	Parameters
Soft tissue	Ogden 1st order	Kallin et al. [68]	μ_1_ = 0.012 MPa α_1_ = 14 d_1_ = 1.67 MPa^−1^
Silicone liner	Yeoh 3rd order	Cagle et al. [44]	c_10_ = 0.02014 MPa c_20_ = −0.00115 MPa c_30_ = 0.00041 MPa d_1_ = 3 MPa^−1^
Socket	Linear–elastic	Plesec et al. [69]	E = 4991 MPa ν = 0.3

**Table 4:** Comprehensive summary of numerical results obtained using a generic transtibial model combined with various liner types.

	Peak Contact Pressure [kPa]			Maximum Deformation [mm]		
PTB Socket	Donning	Heel Strike	Push-off	Donning	Heel Strike	Push-off
Soft	54	127	217	3.8	13.2	17
Medium	74	115	160	3.7	9.7	13.6
Hard	94	153	200	3.5	8.3	11.6
Silicone	66	128	225	3.6	11.2	15.1
TSB Socket						
Soft	63	90	172	5.1	10.3	14.9
Medium	106	129	133	5.1	6.8	10.1
Hard	123	148	160	5.1	5.9	8.2
Silicone	91	119	148	5.0	8.2	11.9

## Data Availability

The original contributions presented in the study are included in the article, further inquiries can be directed to the corresponding author.
